# Flight Planning Optimization of Multiple UAVs for Internet of Things

**DOI:** 10.3390/s21227735

**Published:** 2021-11-20

**Authors:** Lucas Rodrigues, André Riker, Maria Ribeiro, Cristiano Both, Filipe Sousa, Waldir Moreira, Kleber Cardoso, Antonio Oliveira-Jr

**Affiliations:** 1 Institute of Informatics (INF), Universidade Federal de Goiás (UFG), Goiânia 74690-900, Brazil; lsrodrigues@tjgo.jus.br (L.R.); kleber@ufg.br (K.C.); 2 Institute of Exact and Natural Sciences (ICEN), Federal University of Pará, Belém 66075-110, Brazil; afr@ufpa.br; 3 Institute for Systems and Computer Engineering, Technology and Science (INESC-TEC), 4200-465 Porto, Portugal; maria.r.ribeiro@inesctec.pt; 4 Applied Computing Graduate Program, University of Vale do Rio dos Sinos (UNISINOS), São Leopoldo 93022-750, Brazil; cbboth@unisinos.br; 5 Fraunhofer Portugal AICOS, 4200-135 Porto, Portugal; filipe.sousa@fraunhofer.pt (F.S.); waldir.junior@fraunhofer.pt (W.M.)

**Keywords:** Internet of Things (IoT), Unmanned Aerial Vehicle (UAV), autonomous flight planning, optimization

## Abstract

This article presents an approach to autonomous flight planning of Unmanned Aerial Vehicles (UAVs)-Drones as data collectors to the Internet of Things (IoT). We have proposed a model for only one aircraft, as well as for multiple ones. A clustering technique that extends the scope of the number of IoT devices (e.g., sensors) visited by UAVs is also addressed. The flight plan generated from the model focuses on preventing breakdowns due to a lack of battery charge to maximize the number of nodes visited. In addition to the drone autonomous flight planning, a data storage limitation aspect is also considered. We have presented the energy consumption of drones based on the aerodynamic characteristics of the type of aircraft. Simulations show the algorithm’s behavior in generating routes, and the model is evaluated using a reliability metric.

## 1. Introduction

Unmanned Aerial Vehicles (UAVs) are an emerging technology used for many purposes, including military, surveillance, smart cities, and data communication [1,2]. Besides, UAVs (or drones) are becoming more and more popular to provide wireless communication in a vast range of applications and scenarios, including in Internet-of-Things (IoT) environments [3,4,5,6].

When it comes to communication infrastructure, UAVs can be useful to perform data collection on a network of IoT devices [7,8,9]. Data collection from static nodes is a typical application for IoT sensor networks. Given that the sensors are randomly spread over a wide area, it is not easy to obtain information from all sensors if they are not fully connected. The sensors used in an IoT network generally have low available energy. Moreover, some do not have mobile connectivity making the collected data carried out alternatively to the cellular station, either by land or mobile air vehicle. In these cases, UAVs are promising vehicles for data collection in sensor networks due to their direct communication skills between UAVs and the sensor [10].

A UAV has limited autonomy, and the same units are shared among many users, causing their batteries to be found with little remaining charge [11,12]. Lack of battery can risk falling during the flight causing possible loss of this equipment. Another critical aspect to be observed in UAVs is their limiting data storage capacity. The memory provided in these vehicles uses flash technology, which has an ideal weight to carry this type of aircraft. The data storage capacity in drones is a limiting factor because the amount of collected data from the IoT devices can exceed the total storage available, making a mission unfeasible [13,14]. The shared use of the same UAV by several users, coming from different origins, also fills the vehicle’s internal memory capacity, for example, various software installations, storage of collected image files as photos and videos, and other memory used for multiple purposes. Applications of multi-UAVs in the IoT ecosystem have gained great attention. In this regard, Wang et al. [15] proposes a multi-UAV collaborative data collection system, where multiple UAVs collect data from two-dimensional distributed devices in flying mode or hovering mode. Additionally, a multi-UAV deployment for mobile edge computing (MEC) enhanced IoT architecture was designed by Reference [16]. The multiple UAVs are endowed with computing offloading services for ground IoT devices with limited local processing capabilities.

The current scenarios and applications involving UAVs for IoT data collection have shown that the limitations concerning energy and memory must be solved in an integrated way since they represent essential aspects for the flight plan generator. The urge for this integration comes from these limitations, making the problem more challenging and necessary. Moreover, both limitations raise other aspects not addressed by the classic vehicle routing problem. The classic problem described in the literature is called the Vehicle Routing Problem (VRP). Most of the works that focus on VRP do not consider drones acting as data mules. It makes these works inefficient for planning flights aiming for data collection with multiple users and UAVs. Another problem called Vehicle Routing Problem with Drones (VRPD) [17] does not consider the energy and storage limitations when planning drone flights. Due to the lack of works in the literature, it is necessary to improve the existing optimization model responsible for drone flight planning in scenarios involving IoT data collection with multiple users and UAVs.

The main objective of the work is to propose an optimization model for the Vehicle Routing Problem focusing on minimizing the energy consumption and storage limitations. Besides, the proposed model generates flight plans for multiple UAVs avoiding accidents caused by energy shortage and reducing the effects of running out of memory during a flight. Additionally, it is fundamental to highlight that the proposed solution generates a flight plan to be executed autonomously by the aircraft.

The article is organized as follows. Section 2 presents the main related works. Section 3 and Section 4 introduce the formulation of the optimization model and the evaluation results, respectively. Section 5 presents the conclusions of this work.

## 2. Related Work

The UAV-related work is extensive. To discuss the leading research, we start presenting the investigations that use UAVs to collect data from the IoT devices that develop applications to share the aircraft with several users based on cloud services. Therefore, we present the vehicle routing problem with UAVs, and also the works use clustering to group points that will be visited by drones and flight generation approaches.

### 2.1. Applications with UAVs in Wireless Sensor Networks and the IoT Context

To enable a fully automated intelligent transport system in a Smart City context, Menouar et al. [18] used UAVs as IoT devices connected via Dedicated Short Range Communications (DSRC) to cars, providing a Vehicle-to-Vehicle (V2V) and Vehicle-to Communication-Infrastructure (V2X). The work concludes that the truly autonomous operation of UAVs is a real challenge, as it demands the observation of human beings and any other obstacles.

Seiber et al. [19] used drones as IoT sensors to detect hazardous areas contaminated with chemical agents without requiring men to carry out this detection. The communication technology used between the drones was Wi-Fi, and the connection between the drone and the base station was Bluetooth Low Energy (BLE). The limitations found in the tests performed were concerning the scheduled flight of the drones, which was limited by establishing a horizontal alignment in the UAV swarm, which would not happen in a real situation.

Kirichek [20] developed a data delivery model starting from terrestrial sensors, transmitted to drones until it arrives on a cloud server. The proposed model makes it possible to calculate the number of UAVs necessary for data collection and delivery, taking into account the intensity and volume of traffic on the network, the number of units, and the radius of its connection. Jiang and Swindlehurst [21] investigated the optimal trajectory of UAVs equipped with multiple antennas to maximize the sum of transmission rate in uplink communications. Zeng, Zhang, and Lim [22] maximized the throughput of a UAV-based relay system by optimizing the UAV trajectory and the transmission rate between the source and the relay. Mozaffari et al. [23] investigated the optimal deployment and movement of a single UAV to support wireless communications downlink. Although these works present their considerations of the location and trajectory of the aircraft and the best implementation according to aspects of the transmission range and communication technologies used, they still do not consider the data collection function given an IoT sensor network.

A stationary air stop is made to provide a small energy-saving before continuing the UAV movement, without knowing the new trajectory to update the UAV mobility trajectory. This scenario foresees the use of a UAV of medium energy consumption. It considers the maintenance of the drone’s flight for an extended period, making it hover in the air during this period to serve as a base station for the devices. For the scenario determined in Mozaffari et al. [24], situations with autonomy limited to a battery without recharging are considered. However, there are no changes in which devices are active over time, as UAV is used as a data mule, differently from previous work that used a drone as a base station. When drones have low remaining autonomy for flight, they must be used for overflight and data collection from as many IoT devices as possible. In some cases, it is possible to fly over all the existing IoT devices in a smart campus if the autonomy of the drones is used efficiently.

Yang and Yoo [10] first acquired data sensing points from the entire sensor field. UAV communicates with the sensors to obtain data, then determine the best trajectory to traverse the neighboring acquisition points. A genetic algorithm is used to perform the optimization. In a similar context, Sujit et al. [25] UAV flew over sensors retrieving data. To minimize UAV flight time and maximize network lifetime, a joint optimized route for UAV and sensor network was executed. The main technique used to make this possible is clustering. Despite achieving excellent results, neither work establishes this optimal trajectory that demonstrates an energy expenditure model based on drone aerodynamics.

### 2.2. Cloud Drone Sharing Services

Choi et al. [26] used a standards-based communication scheme that is useful in realizing efficient management and control of UAV-based operations. Considering this issue, they used a global standard called one Machine-to-Machine (M2M), which provides M2M communication and a service layer to interoperate M2M and IoT solutions.

Koubâa et al. [27] contributed to the Internet of Drones (IoD) and the deployment of UAVs over the cloud. An innovative service-oriented and cloud-based system and a management system provided access to UAVs through Web Services, scheduling missions, and promoting collaborations between drones.

In both works, the services developed do not include the drone’s availability when flying over a network of IoT sensors. This service attends to numerous users or client applications that define what sensors they need to consume data.

### 2.3. Vehicle Routing Problem with UAVs

Isaacs et al. [28] exploited data collector mobility to mitigate the power consumption of stationary nodes and enable sparse deployments at the cost of additional data latency. Moreover, they looked for efficient strategies to alleviate this data latency. They also provided data mule routing policies for locating acoustic sources in a large area involving many sources and sensors. Fügenschuh et al. [29] formulated the mission planning problem for a fleet of UAVs as a Mixed Integer Non-Linear Programming (LMLP) problem. The problem calls for a selection of targets from a list for the UAVs and trajectories that visit the chosen targets.

Semiz et al. [30] developed an algorithmic method to solve the vehicle routing problem applied to UAVs. This method uses the divide and conquers method for resolution. In this way, the problem is transformed into a combination of several sub-problems. A method is designed to convert these minor problems into transport problems. Each transport problem is solved with the simplex algorithm.

Robotic aerial systems can be beneficial to perform complex tasks in a distributed and cooperative manner, such as locating targets and searching for Points of Interest (POIs). Guerriero et al. [31] proposed a distributed autonomous UAV system capable of coordinating and cooperating to ensure spatial and temporal coverage of specific time and variable spatial POIs. It is considered that the UAV system can solve distributed dynamic scheduling problems, requiring each device to move towards a certain position at a certain time.

VRPD is an extension of the classic capable vehicle routing problem, where trucks and drones are used to deliver to customers. A distinctive feature of the VRPD is that a drone can travel with a truck, take it away from its stop to service customers, and land at a service center to travel with another truck as long as range and carrying capacity limitations are met. Wang et al. proposed a mixed-integer programming model and develop an algorithm using the branch-and-price method [17]. In a similar approach, Adbelhafiz et al. briefly analyzed VRP instances for UAV operations with multiple objectives [32]. Moreover, the authors focus on multi-objective and multi-UAV mission planning problems and try to take advantage of the literature on VRP and its variants. The results show that each military multi-UAV mission has its corresponding VRP variant. A new algorithm based on a tree search enhancement to solve complex multi-UAV mission planning problems with complex constraints is presented.

Dorling et al. proposed two multi-trip VRPs for delivery *drones* addressing multiple trips to the depot and the effect of battery weight and payload on energy consumption [33]. One minimizes costs constrained to a delivery time, while the other minimizes delivery time subject to a budget constraint. The authors propose a power consumption model for multi-rotor drones, demonstrating that power consumption varies approximately linearly with payload and battery weight. In this similar scenarios, Coutinho et al. [34] defined the UAV Routing and Trajectory Optimization Problem. A taxonomy was introduced, and recent contributions to optimizing the UAV trajectory, UAV routing, and article approach to these problems and their variants were reviewed.

The use of only one class of algorithm to solve complex problems, such as data mule routing, has proven not to be a good strategy, so the above works use chaining algorithms to have the best processing performance and the best optimal solution. However, the authors do not make use of clustering in this thread to achieve the optimal result. MILP can accommodate both continuous and binary decision variables and a variety of constraints and objective functions. However, the NP-Complete nature implies a dramatic increase in computation complexity as the number of variables and constraints increases. This issue is highly critical and needs to be resolved in near real-time.

### 2.4. Trajectory Optimization Methods

Trajectory optimization methods that take aircraft dynamics and path constraints is very relevant for problems addressed in this work. Hence, Hong et al. [35] proposed a sequential quadratic programming (SQP) based optimization for state and input constrained guidance of aerospace vehicles with a free final time. The idea is to reformulate a class of nonlinear guidance with path and terminal constraints into a generic quadratic programming (QP) and then to solve problems of the SQP concept. In this regard, a scheme with five foundational elements is developed and tailored to the requirements of missions by Reference [36].

Additionally, a very recent and relevant work proposed by Hong et al. [37] proposed a hierarchical parameterization method in order to generating smooth flight trajectories satisfying hard boundary constraints on controls and control derivatives of all orders. The proposed method is to achieve a seamless transition that accurately connects two steady flights. These methods are relevant for generating trajectories between sensors for the vehicles.

Wang et al. [38] proposed a communication scheme based on UAV-enabled wireless powered communication network (WPCN) that can improve the energy harvesting efficiency. Authors have used the UAV trajectory, time allocation, transmit power, scheduling of wireless information, and wireless power transfer to minimize the whole energy consumption.

In summary, methods that carry out exhaustive research may become unfeasible as the sample space increases. Our proposed models have two different and complementary goals, (i) to maximize the total number of visited sensors and (ii) to achieve the minimum energy consumption for that maximum number of sensors. The next section is dedicated to our proposal.

## 3. Flight Plan Optimization of Multiple UAVs with Storage Capacity

This section presents the formulation of our proposed optimization model, including the aspects not covered by the classical formulations based on VRP. This section is organized into Problem Formulation (Section 3.1) and Hovering Calculation (Section 3.2). We highlight that our proposed model does not consider the collisions of any types.

### 3.1. Problem Formulation

Let the set K={1,2,…,n} represent the drones used to carry out the mission to be optimized. Consider a set of clusters S={0,1,2,…,n} of sensors, forming an IoT network. These sensors are scattered around campus at random, establishing an arc of the distance between each of these sensors. The set *S* has the number of elements equal to the number of sensors added to the starting point for modeling purposes. The element 0 is a virtual node that identifies the location from where the aircraft takes off. Moreover, we consider that drones can have heterogeneous characteristics, and the energy consumption between the sensors is represented in this case by the set $E=\{E_{0,0}^{1},E_{0,1}^{1},\ldots ,E_{n-1,n}^{k}\}$, in this set, an element $E_{i,j}^{k}$ has i,j∈S, and k∈K. In the case of i=j, the energy cost is zero. Each drone that performs the flight in this trajectory has a specific autonomy at the moment before the flight. In this context, we present the set $C=\{C_{1},C_{2},\ldots ,C_{n}\}$, and each element $C_{k}$ represents the specific autonomy of the UAV *k*. The set shown above is also known as VRPD since the objective of the classic model is to minimize the distance traveled by vehicles. However, in this work, as a form of prevention, the objective is to maximize the number of visited sensors. In this case, it is necessary to restrict the total energy expenditure of each vehicle to the autonomy that each one has.

The capacity attribute of the problem statement is treated as the data storage capacity. Most commercial drones that are on the market are sold with attached flash memory. These memories have a high cost and are provided with small storage space. Furthermore, they are used because their weight, being small, does not influence the total weight of the drone, helping the vehicle maintain its reasonable total range. Sensors or sensor clusters have a maximum storage buffer for the data that will be collected. In this case, we present the set $q=\{q_{1},q_{1},\ldots ,q_{n}\}$, where each element $q_{s}$ represents the total buffer in each specific cluster. To identify the storage capacity of the flash memory that the UAV loads, we have the set $Q=\{Q_{1},Q_{1},...,Q_{n}\}$ for each aircraft. For each of the segments computed in the flight routing, it is necessary to determine a binary variable that specifies whether the edge (i,j) will be covered by the drone *k*. This decision variable is given in (1):

(1) $$X_{ij}^{k}\in \left\{0,1\right\},\forall i,j\in S,\forall k\in K.$$

The most significant interest is that as many sensors or clusters can be visited, considering that vehicles have battery limitations. Moreover, UAVs must take off from the first point and end the flight returning to the same point for this scenario. To know the number of visited points, given a set $A_{r}$ of traveled edges, it is necessary to subtract $A_{r}-k$, as the last edges traveled are relative to the drones’ turn to the first point. The objective of the optimization model is to maximize the number of visited points by maximizing the sum of covered edges. This objective is demonstrated in (2):

(2) $$Maximize\sum_{i,j\in S,k\in K}X_{ij}^{k}.$$

Constraint (3) ensures that the sum of times a given UAV arrives at a point through an edge. For example, (i,j) must be equal to the sum of the output edges of the same point, i.e., (j,i), this output being executed by the same drone that executed the input. This restriction is necessary because, in each sensor or cluster that a drone enters, overflying does not initially allow another vehicle to visit that point. In this case, an exit path is an edge from the last point to the next sensor point to be visited.

(3) $$\sum_{i\in S,i\neq j}X_{ij}^{k}=\sum_{i\in S,i\neq j}X_{ji}^{k},\forall j\in S,\forall k\in K.$$

Constraint (4) and (5) determine that each vehicle must leave the starting point exactly once and return once.

(4) $$\sum_{i\in S,i\neq 0}X_{0i}^{k}=1,\forall k\in K,$$

(5) $$\sum_{i\in S,i\neq 0}X_{i0}^{k}=1,\forall k\in K.$$

Constraint (6) gives the possibility that a point is overflown. In this scenario, not all points are visited, considering that the vehicle does not have the autonomy to do so. However, there is a limit of visitation at most once. It is essential to highlight that the VRP classic considers it mandatory to visit all points.

(6) $$\sum_{k\in K}X_{ij}^{k}⩽1,\forall i,j\in S,i\neq j.$$

For the drone to store the data collected from all visited clusters, the total amount of data collected by a specific UAV must be less than or equal to the storage available on that drone as formalized by restriction (7). The total energy consumption when visiting the points and hovering to perform the synchronization must be less than the autonomy in the specific vehicle (8). The $e_{ij}^{k}$ is the energy spent by UAV to travel between points ij. The $C_{k}$ is the total energy capacity for power consumption.

(7) $$\sum_{i,j\in S,i\neq j}q_{j}X_{ij}^{k}\leq Q_{k},\forall k\in K,$$

(8) $$\sum_{i,j\in S,i\neq j}e_{ij}^{k}X_{ij}^{k}\leq C_{k},\forall k\in K.$$

Given a subset *R* of clusters within the total set, in this one, the sum of edges traveled must be equal to the total of vertices minus one so that a subpath is not closed within that set of clusters (9). This restriction acts to avoid any waste in the remaining autonomy of the drone during the flight. Considering if a sensor has already been visited before, it is not necessary to return to it.

(9) $$\sum_{k\in K}\sum_{i,j\in S,k\in K}X_{ji}^{k}\leq \left|R\right|-1,R\subseteq K.$$

### 3.2. UAV Hovering Calculation

This section describes the calculation of power consumption during the hover to use in the optimization problem. The technology used was BLE, which has an Air Data Rate of 1Mb/s for the connection between UAV and sensors [39]. Let set $H=\{H_{1},H_{1},...,H_{n}\}$ represent the values related to the energy consumption of the drone during the planning in which the data contained in the buffer of the sensors or clusters is transmitted.

The calculation of power consumption during the drone’s specific hover is shown below. The Buffer is divided by the Data Rate to determine how long it takes to transmit the entire buffer collected by the sensors. Right after that, to measure the energy consumption of the plantation, the classic energy formula is used: E=P×t. This set of values resulting from energy consumption from the moment of data transmission are added to the data consumption values for visiting at a given point, making the optimization model consider all energy expenditure as a single set.

**Figure 1:**
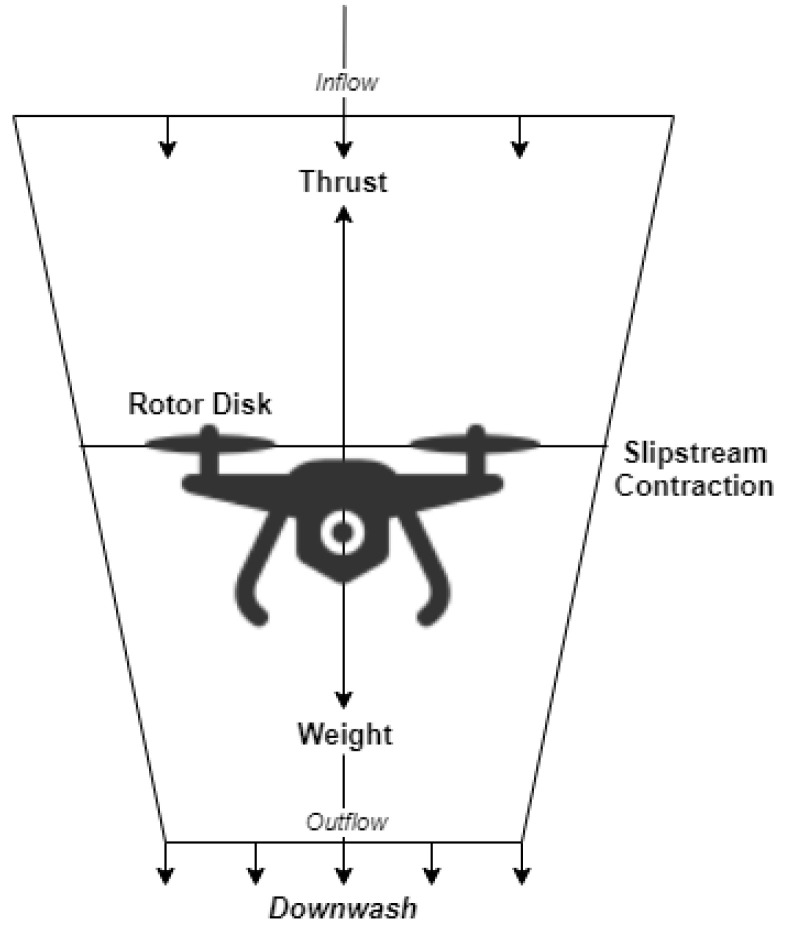
Hover condition with flow tube around rotor disc and frame.

The simplest method to obtain a first-order estimate of the power needed for the hover is the One-Dimensional Axial Momentum Theory [40]. The slipstream has a vertical axis through the center of the rotor. The inflow is from the top of Figure 1. Slipstream contraction is explained with the same concepts used for the helix. When we take into account these changes for application to the suspended helicopter, the One-Dimensional Axial Momentum Theory provides the induced speed in the hover engine (10). The *W* means weight, *T* means engine thrust, and ρ means air density:

(10) $$V_{h}=\sqrt{\frac{T}{2\rho A}}=\sqrt{\frac{W}{2\rho A}}.$$

The product gives the ideal power needed to hover between the thrust and the induced speed, that is, (11):

(11) $$P_{h}=T\times V_{h}=W\sqrt{\frac{W}{2\rho A}}=\frac{W^{\frac{3}{2}}}{\sqrt{2\rho A}}=\sqrt{\frac{W}{2\rho }}\sqrt{\frac{W}{A}},$$

where the disk area is $A=\pi R^{2}$, we conclude that the induced power to hover increases with the aircraft’s weight and decreases with increasing rotor radius (all other parameters being constants). This assumption would imply that increasing the diameter would benefit considering constant weight, which is not always the case.

## 4. Evaluation and Results

We have used MATLAB and PuLP library of Python to evaluate our proposed optimization model. PuLP is an open-source linear programming (LP) package which largely uses Python syntax and comes packaged with many industry-standard solvers. Simulations presented in this section, two UAVs of equal storage capacity were considered, with 4000 mb each. The values of 5, 7, 9, and 11 points were simulated. Moreover, we define that each vehicle had a total autonomy of 30,000 J of energy to be spent.

**Figure 2:**
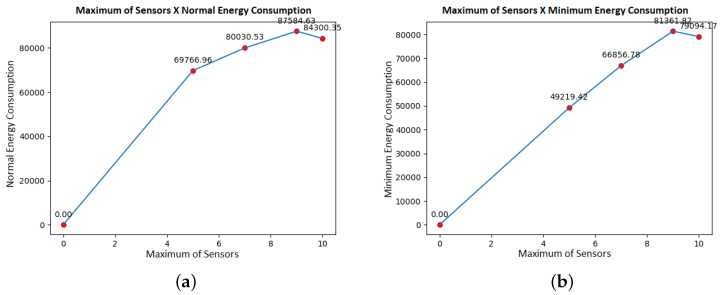
Maximum of sensors versus energy consumption. (**a**) Normal energy consumption. (**b**) Minimum energy consumption.

It is essential to highlight that, in Figure 2, the energy consumption changes and is reduced using the proposed optimization model. One optimization objective is to minimize the energy consumption of as many sensors as possible to be visited. At the last point of the graphs, it is possible to see that there is a similar drop in consumption in the illustrations. This drop happens because, even if the number of sensors visited is more significant than in the previous simulated set, more possibilities to minimize power consumption are created as the sample space increases.

**Figure 3:**
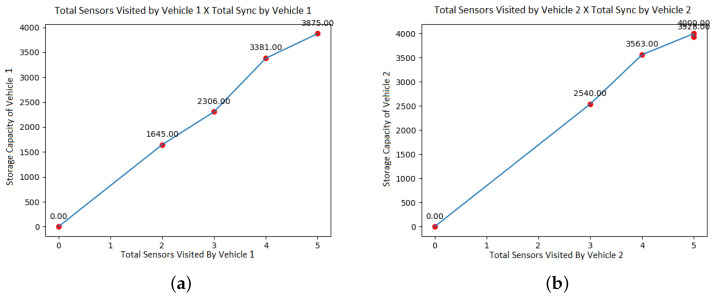
Total sensors visited versus total sync. (**a**) Total sensors visited by Vehicle 1. (**b**) Total sensors visited by Vehicle 2.

We can see in Figure 3 that the amount of data collected has an almost linear growth and has an approximately equal distribution between the two vehicles. This behavior occurs because both the load and the storage capacity of the two UAVs are set with equal values.

In Figure 3, the total data collected by each of the vehicles is compared. As the number of simulated nodes increases, the volume of data naturally also increases. Although UAV 2 presents a greater volume of data collected than UAV 1, there is no weighting in the model that causes this, and the storage capacity of the two vehicles is equal.

**Figure 4:**
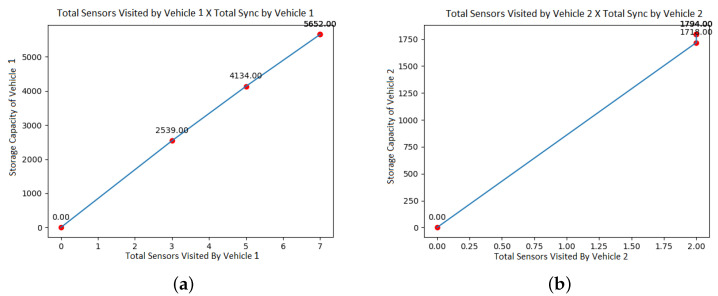
Total sensors visited versus total sync. (**a**) Total sensors visited by Vehicle 1. (**b**) Total sensors visited by Vehicle 2.

Following the approach, a new simulation is made, as shown in Figure 4 keeping all the previous data but changing only the total storage capacity. In this case, UAV 1 now has 6000 mb of space, and UAV 2 now has 2000 mb of space. We highlight that the model makes the proper distribution in routing so that each vehicle does not overload your storage space.

**Figure 5:**
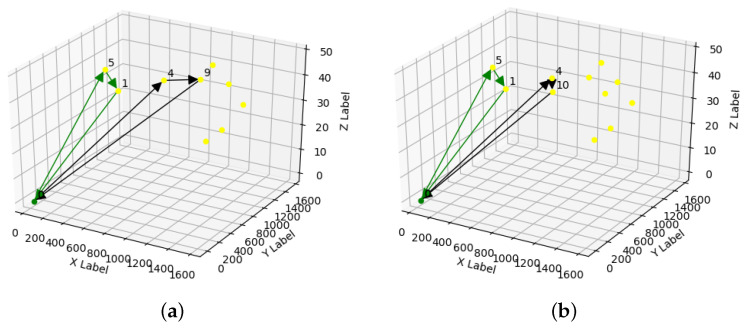
Routes with equal and different storage capacity. (**a**) Route with 9 points. (**b**) Route with 11 points.

Figure 5 present the routes generated by the previous cases when the storage capacity was similar. We can observe that the amount of visited sensors also remains the same between the two vehicles. However, we observe that, despite the significant variation in storage capacity, the number of sensors between the two does not differ that much. This behavior happens because the model only selects an optimal solution that we define with a larger buffer size.

**Figure 6:**
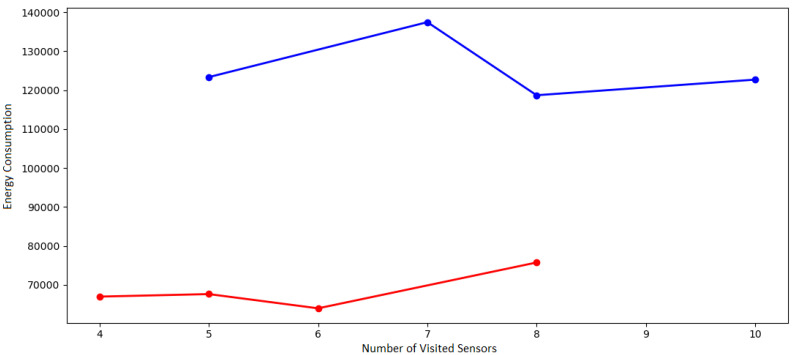
Comparison between the model with a single vehicle and multi vehicles.

Figure 6 presents 4 instances which were simulated allocating 5, 7, 9, and 11 sensors. Each instance is run simultaneously in the multi-vehicle model and the same in the single-vehicle model. In this comparison, the same range of 80,000 J is attributed to each vehicle. It is possible to observe the natural findings that, in the model with multiple vehicles, considering that the instance uses two aircraft, it is possible to fly over more sensors. However, the energy expenditure is necessarily higher. In this case, because the model requires the two aircraft to leave and return at least once, an improvement would be the possibility that the model assigns the tasks to only one UAV. In this case, if it identifies that this way, it would use a lower total of energy.

### Model Performance

We have used response time, reliability rate (with variation in autonomy and visitation points) and also the amount of energy consumption to extract data in order to analyze the model’s performance with the confidence interval of 95% during the simulations.

**Figure 7:**
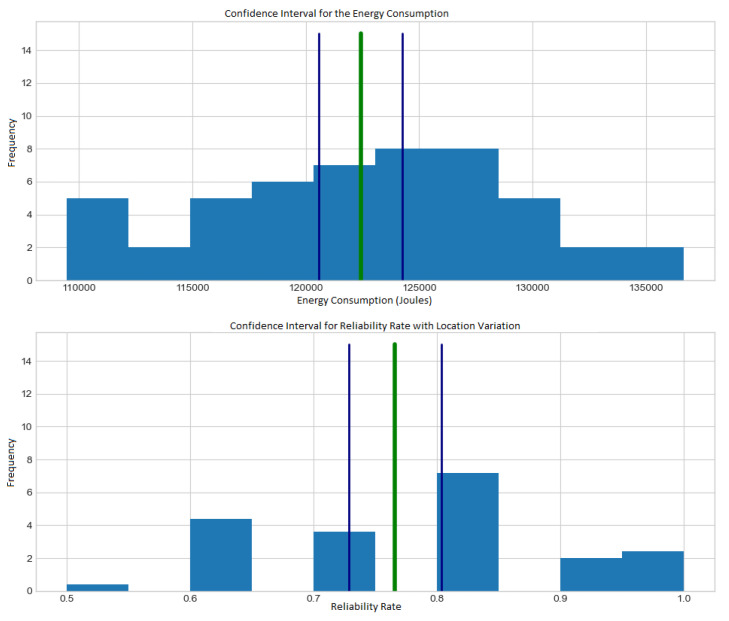
Histogram with energy consumption and reliability rate varying locations.

**Figure 8:**
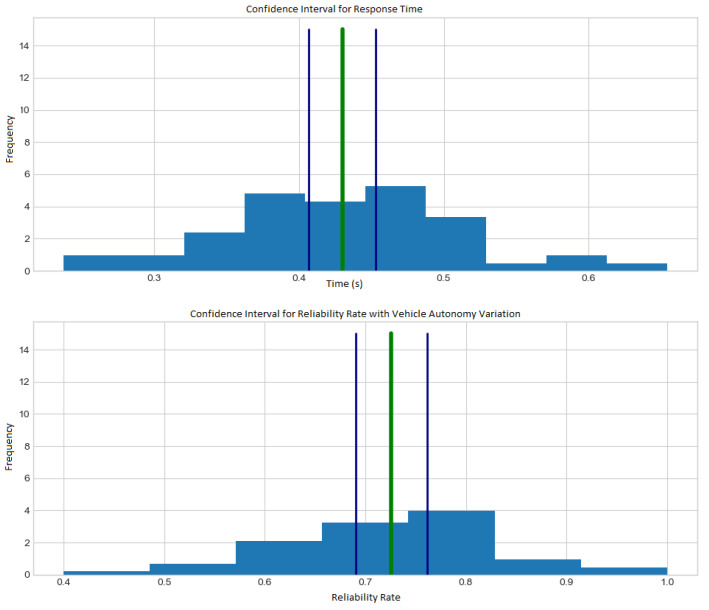
Histograms with response time and reliability ratio varying autonomy.

Figure 7 show the histogram with energy consumption and reliability rate (with variation in visitation points). Figure 8 show the histogram with response time and reliability rate (with variation in autonomy).

For the reliability rate, in the two cases used, it is given as shown in Equation (12) [41] where *R* is the number of replications of the simulation, and *n* the number of failures, that is, the model does not have an optimal solution for the input data.

(12) $$r=\frac{R-n}{R}.$$

According to Figure 7 and Figure 8, the situation can happen because the basic premise is that each vehicle must leave and return to the starting point at least once. However, there may be situations in which the vehicle will not have enough autonomy to perform such tasks so that the simulation will show no doable results. From other replications of the simulations, 50 samples of mean values are extracted. Each of the samples is taken from a simulation run from a different instance, considering new sensor location points and variations in vehicle ranges.

A constant autonomy is maintained, allowing all instances to return an optimal solution to measure the response time and energy consumption metrics. For simulations with autonomy variation, a random value is generated between 35,000 and 60,000 joules.

(13) $${I.C.}_{media}:\mu \pm \left(t_{\left(1-\frac{\alpha }{2},df\right)}\right)\times \frac{s}{\sqrt{n}}.$$

According to Figure 7 and Figure 8, the confidence interval formula for the sample mean is used confirm shows Equation (13), where μ is the arithmetic mean of the samples, and *t* is the *t-distribution* function, which depends on α, called significance level, and on df which is the level of freedom. The element *s* is the sample variance, and the *n* is the sample set size.

## 5. Conclusions

We have proposed a routing optimization model for multiple UAVs as a Data Mule that considers the stopping time for data transfer, vehicle aerodynamics, drone autonomy, buffer size of IoT devices, and UAV storage capacity. The model differs from the literature because it focuses on preventing crashes due to lack of battery during the flight. This preventing crash entails a series of modifications in the model compared to the traditional structure of the vehicle routing problem.

The concern with storage capacity is also a fundamental differential in the model as drones remain a scarce and limited resource in organizations. Autonomous and scheduled flights must be concerned with the capacity of the data allocation space as this resource will be used by several users. When flying over clusters, UAVs can encounter a heavy mass of data. Another fundamental aspect was the calculation of energy expenditure during data transmission at each stop.

As a future work, we are carrying out real experiments using the bebop2 by Parrot drone. We are also considering applying our model with energy harvesting following the work done by Reference [38]. Another issue is that the bit rate must be calculated for the transmission of data each to from a dynamic model. There are some elements that can cause signal degradation and that can impact this transmission rate, such as path loss and interference from simultaneous connections among each other. The air vehicle may encounter a group of sensors with heterogeneous communication technologies, as well as a number of nodes, that it does not have the capacity to make all connections.

## Data Availability

Not applicable.
